# Wastewater as an early indicator for short-term forecasting COVID-19 hospitalization in Germany

**DOI:** 10.1186/s12889-025-24149-2

**Published:** 2025-08-25

**Authors:** Jonas Radermacher, Steffen Thiel, Aimo Kannt, Holger Fröhlich

**Affiliations:** 1 Department of Bioinformatics, Fraunhofer Institute for Algorithms and Scientific Computing (SCAI), Schloss Birlinghoven 1, Sankt Augustin, 53757 Germany; 2 University of Applied Sciences Bonn-Rhein-Sieg, Sankt Augustin, Germany; 3 Fraunhofer Institute of Translational Medicine and Pharmacology ITMP, Theodor-Stern-Kai 7, Frankfurt am Main, 60596 Germany; 4 Institute of Clinical Pharmacology, Faculty of Medicine, Goethe University Frankfurt, Theodor-Stern-Kai 7, Frankfurt am Main, 60590 Germany; 5 Bonn-Aachen International Center for IT, University of Bonn, Friedrich Hirzebruch-Allee 6, Bonn, 53115 Germany

**Keywords:** Pandemic, COVID-19, Wastewater, Surveillance, Machine Learning, Forecasting

## Abstract

**Background:**

The COVID-19 pandemic has profoundly affected daily life and posed significant challenges for politics, the economy, and the education system. To better prepare for such situations and implement effective measures, it is crucial to accurately assess, monitor, and forecast the progression of a pandemic. This study examines the potential of integrating wastewater surveillance data to enhance an autoregressive COVID-19 forecasting model for Germany and its federal states.

**Methods:**

First, we explore the cross-correlations between SARS-CoV-2 viral RNA load measured in wastewater and COVID-19 hospitalization considering different time-lags. Further, the study compares the performance of different models, including Random Forest regressors, XGBoost regressors, ARIMA models, linear regression, and ridge regression models, both with and without the use of wastewater data as predictors. For decision tree-based models, we also analyze the performance of fully cross-modal models that rely solely on viral load measurements to predict COVID-19 hospitalization rates.

**Results:**

Our retrospective analysis suggest that wastewater data can potentially serve as an early warning indicator of impending trends in hospitalization at a national level, as it shows a strong correlation with hospitalization figures of up to 86% and tends to lead them by up to 8 days. Despite this, including wastewater data in the prediction models did not statistical significantly enhance the accuracy of COVID-19 hospitalization forecasts. The ARIMA model without the inclusion of wastewater viral load data emerged as the best-performing model, achieving a Mean Absolute Percentage Error of 4.76% forecasting hospitalization 7 days ahead. However, wastewater viral load proved to be a valuable standalone predictor, offering an objective alternative to classical surveillance methods for monitoring pandemic trends.

**Conclusion:**

This study reinforces the potential of wastewater surveillance as an early warning tool for COVID-19 hospitalizations in Germany. While strong correlations were observed, the integration of wastewater data into predictive models did not improve their performance. Nevertheless, wastewater viral load serves as a valuable indicator for monitoring pandemic trends, suggesting its utility in public health surveillance and resource allocation. Further research may help to clarify the real-time applicability of wastewater data and expand its use to other pathogens and data sources.

**Supplementary Information:**

The online version contains supplementary material available at 10.1186/s12889-025-24149-2.

## Background

The COVID-19 pandemic has significantly disrupted global society, impacting healthcare systems, economies, and daily life on a worldwide scale. This global crisis has revealed weaknesses in healthcare infrastructure and underlined the necessity for reliable predictive tools to effectively manage future pandemics [1]. Accurate forecasting of incident cases and hospitalization is essential for optimizing resource allocation or decision making in particular for potential future pandemics. Many approaches in this regard have been published during the COVID-19 pandemic or even before [2].

One approach to pandemic monitoring is wastewater surveillance, which has emerged as a promising method for early detection of viral spread of COVID-19 [3]. Wastewater data can provide a non-invasive, community-level indicator of infection prevalence, potentially offering a lead time advantage over incident cases, hospitalizations, and deaths [4, 5]. Olesen et al. reviewed various studies investigating the lead time of wastewater-based epidemiology for COVID-19 and reported lead times of up to 14 days with respect to newly reported cases [6]. Another advantage of wastewater surveillance is that it does not depend on individual testing behavior and availabilities and that it is not biased towards severe cases leading to hospitalization and on the other hand can capture both symptomatic and asymptomatic infections [7]. A disadvantage of wastewater surveillance can be the change in relationship between the viral load, true infections and classical surveillance, for example due to different pathogen variants or population immunity. This could lead to inaccurate detections and false conclusions [8]. Historically, wastewater assessment was already performed in the 1960 s for Polio [9]. It was shown that wastewater data could be utilized for early warning of pandemic outbreaks and new emerging variants of concern [10–13], and it was also used as a predictor for incident cases and hospitalization [14–16]. Some of these studies also investigated these aspects in specific countries, including Germany [10, 12, 13, 16]. However, these studies either focused on specific regions or failed to explore the potential predictive power of wastewater viral load in the context of short-term pandemic forecasting, particularly concerning hospitalization. Short-term forecasting is particularly important for accurate resource planning, e.g. ICU beds, ventilators, or planned surgeries while being able to adapt to dynamic changing conditions, due to for instance vaccination, non-pharmaceutical interventions (NPIs), and new emerging variants.

This study aims to address this gap by examining the relationship between SARS-CoV-2 viral RNA loads measured in wastewater and COVID-19 hospitalization across Germany at a regional and national level. Further, we evaluate the potential of integrating wastewater data into various autoregressive models, such as Random Forest, XGBoost, ARIMA, linear regression, and ridge regression models, to improve their predictive accuracy for forecasting hospitalization in Germany. Furthermore, we investigate the efficacy of models that utilize only wastewater data in predicting hospitalization. These cross-modal models can be particularly helpful when the indicator of interest (here hospitalization) is not recorded or suffers from large reporting delays. To our knowledge, the potential of wastewater data used in this study has previously not been explored for predictive modeling.

## Methods

### Surveillance data

We utilized two primary datasets to investigate the relationship between wastewater surveillance data (SARS-CoV-2 RNA) and COVID-19 hospitalization in Germany.

The first dataset contains the daily incident hospital admissions (reported by all hospitals across Germany), which were aggregated by the Robert Koch Institute (RKI). In this dataset, the hospital admissions are represented as the 7-day hospitalization normalized to 100k people (rate) [17]. 7-day hospitalization here corresponds to the one-sided moving average over the past 7 days of recorded hospitalizations. The moving average preserves the daily time resolution and smoothens the data, reducing short-term fluctuations. In the following, we will refer to this data as hospitalization rates. One key metric frequently utilized during the pandemic was the number of reported incident cases. However, this metric is heavily influenced by the availability of testing, testing strategies, and underreporting (often referred to as the ‘dark figure’). Therefore, we considered hospitalization to be a more reliable metric and excluded incident cases from the subsequent analysis.

The second dataset used in this study is the wastewater viral load which is based on measurements collected from sewage treatment plants participating in the AMELAG (Abwassermonitoring für die epidemiologische Lagebewertung) project, coordinated by the Robert Koch Institute (RKI) and the Federal Environment Agency (UBA). This project aims to provide a robust, harmonized surveillance infrastructure for monitoring the spread of infectious diseases at the population level through wastewater analysis [18]. In the following, we will describe the sampling, analysis, and processing. More information can be found in the technical guidelines [19–22].

Wastewater samples were collected from up to 168 sites across Germany as of 2024. These plants are geographically distributed to cover a substantial proportion of the German population, ensuring representativeness for national surveillance. Sampling is performed twice weekly, typically on Mondays and Wednesdays, following standardized protocols to ensure the comparability and reliability of the data. Specifically, automated samplers collect hourly aliquots (0.5 L per hour) of raw influent wastewater over a 24-hour period, which are then homogenized into a single 1-liter composite sample per plant per sampling day. This method is designed to capture daily fluctuations in wastewater composition and provide a representative measure of the viral load entering each facility. During collection and transport, samples are kept cooled and are processed within 48 h to preserve RNA integrity.

At the participating laboratories, the samples undergo a standardized processing workflow. First, solids are removed by centrifugation (typically at 4000–5000 x g). Viral RNA is then concentrated from the liquid fraction using polyethylene glycol precipitation or ultrafiltration. Detection and quantification of viral RNA are performed using reverse transcription quantitative polymerase chain reaction (RT-qPCR). The primary targets for quantification are SARS-CoV-2. In SARS-CoV-2 testing, single PCR reaction targets are genes from the nucleocapsid (N1, N2), envelope E, and polymerase (RdRp), creating redundancy to help offset potential errors. Since 2024, the protocol has also included influenza A/B and respiratory syncytial virus (RSV). In this study, we will focus on SARS-CoV-2. Comparative studies and inter-laboratory quality assessments are regularly conducted to confirm the comparability of results and to determine detection limits for each pathogen.

A crucial step in the processing of the wastewater data is normalization. The measured viral loads are normalized to account for fluctuations in wastewater volume and composition, which can be influenced by factors such as rainfall or population size. The normalization method adjusts viral concentrations based on the ratio of the plant’s current inflow to its average dry-weather inflow, according to the formula:

$$\begin{aligned}\mathrm{Normalized}\;\mathrm{Viral}\;\mathrm{Load}\;=&\;\mathrm{Raw}\;\mathrm{Viral}\;\mathrm{Load} \\&\times\;\frac{Q_{\mathit c\mathit u\mathit r\mathit r\mathit e\mathit n\mathit t}}{Q_{\mathit m\mathit e\mathit d\mathit i\mathit a\mathit n}}\end{aligned}$$

Where Q_median_ is the average dry-weather inflow, and Q_current_ is the inflow during the sampling period and Raw Viral Load corresponds to the geometric mean of the PCR values of both samples. The result is then one normalized viral load value per week which is linked to the date of the week’s Wednesday. Additional normalization parameters, such as surrogate viruses (e.g., PMMoV, CrAssphage) and trace substances, are also being evaluated for their potential to further improve the accuracy of population-level estimates but are not current practice.

Data from multiple treatment plants are then aggregated to provide regional and national overviews. Aggregation is performed using population weighting to ensure that each plant’s contribution reflects the size of the population it serves.

The wastewater data had not been collected or at least published before June 2022. Therefore, we restricted ourselves to the timeframe of June 2022 to the end of 2023, covering 82 weeks, in which all datasets were available. In the AMELAG project a symmetric LOESS [23] is used to obtain daily smooth-curve estimates. Because of the symmetric window applied, future data is needed, which in a real-time scenario would not be possible. Therefore, we followed a similar approach but restricted our self to a one-sided LOESS, using the past 4 weeks of data points for interpolation and smoothing. LOESS, in contrast to the moving average applied to the hospitalization data, is applied to both interpolate intermediate daily values and smooth the resulting curve, making it well-suited for increasing the data’s temporal resolution.

We decided to not go beyond the year 2023, as the WHO had declared an end to COVID-19 as a Public Health Emergency of International Concern (PHEIC) already on May 5, 2023 [24]. With this the recording of incident cases, hospitalizations, and deaths decreased. Still, during the winter season at the end of 2023, rising numbers were observed. Because of this and also to cover two winter seasons and with that a more representative time frame, we opted to include data up to the end of 2023.

A total of 135 unique treatment plants participated in the AMELAG study, with the number of active plants ranging from 28 to 133 during the study period. The smallest treatment plant (Glonn, Bayern) served a population of 5,300, while the largest (Berlin) was connected to 1.5 million people. The median population served per plant was 95,000, and the average was approximately 185,000. The total population connected to the treatment plants ranged from about 9.5 million to 25 million. Table S2 provides a comprehensive overview of the treatment plants, including details on the sampling periods and the populations served. Additionally, Table S3 offers a breakdown of the number of treatment plants per state and per week, as well as the total number of treatment plants and the total connected population for each week.

### Cross-correlation analysis

Our first step was to investigate the cross-correlation between viral load in wastewater data and country-level hospitalization rates. We normalized both the hospitalization rates and wastewater data using a min-max normalization. After visually inspecting the normalized time series, we employed cross-correlation analyses to evaluate the relationship between wastewater viral load (both weekly and daily) and hospitalization rates. For the visualization we used the smoothed hospitalization rates and interpolated viral load data, the cross-correlation analysis was performed using both, the raw time series and the smoothed ones. For the cross-correlation, we calculated Spearman [25] correlation coefficients. Spearman coefficients evaluate monotonic relationships, which can be particularly useful when marginal distributions of individual variables do not meet the assumptions of normality. Our hypothesis was that there exists consistently a time lag between the two variables, with trends in wastewater viral load preceding similar trends in hospitalization rates. This is based on the premise that viral load in wastewater increases or decreases several days before individuals exhibit symptoms severe enough to require hospitalization. Consequently, we performed a lagged cross-correlation analysis to explore this temporal relationship, covering lag values between − 7 and 14 days. We shifted the wastewater and hospitalization time series relative to each other to identify the highest positive correlation and, consequently, the time lag at which the wastewater data could most effectively predict hospitalization. Although the original wastewater viral load data is collected on a weekly basis, it is reported as the value for each week’s Wednesday, effectively providing a daily estimate for that day. In practice, wastewater samples are collected twice a week, on Monday and Wednesday. The PCR results from both days are combined using a geometric mean, and this aggregated value is then normalized according to the flow rate. As a result, the reported Wednesday value is not a measurement for a single day, but rather an average reflecting conditions across both Monday and Wednesday. This introduces an uncertainty of up to two days in the exact timing of the wastewater measurement. When calculating the cross-correlation between two time series, one with daily resolution (e.g., hospitalizations) and one with weekly (e.g., wastewater data), the correlation is computed only for days when both series have data. For a lag of 0 days, the correlation is calculated between all Wednesday values in both series; for a lag of 1 day, between Wednesday wastewater values and Thursday hospitalization values, and so on. This approach allows us to estimate the time delay between the two series at a daily resolution, but it is important to consider the inherent timing uncertainty in the wastewater data due to its aggregation process.

### Forecasting models

In the following part we wanted to assess whether the inclusion of the wastewater viral load into otherwise autoregressive models, will significantly improve the model forecasting performances.

We employed three classical models: linear regression, ridge regression, and ARIMA, and two decision tree-based ones: XGBoost and Random Forest. These methods were chosen because they proved useful in our previous study [26]. The objective of all models was to forecast the daily country-level hospitalization rates seven days ahead. Only ARIMA is per definition an autoregressive model, meaning it uses past and present data of one indicator to predict future data of this exact indicator. However, other models can be used in an autoregressive manner as well, simply by using past and present data of the variable to predict as sole input as well.

### Data transformation

Given the exponential growth/decay patterns in hospitalization rates and wastewater viral load (Fig. 3), we applied a log-transformation to both time series. This transformation linearizes exponential trends, aligning with the additive assumptions of linear and ridge regression models explained later. Further, log-transformation can help stabilize the variance [27]. For the tree-based models, log-transformation is not necessary, as these algorithms can handle non-linear relationships inherently [28, 29]. However, it may still simplify the modeling task by reducing the dynamic range of the data, potentially improving the model performance. For all forecasting models we used the smoothed hospitalization rates and the interpolated viral load data, aligning both time series and reducing noise.

### Linear regression and ridge regression

For our linear regression model, we employed the LinearRegression class from scikit-learn [30], specifically version 1.3.2. The general equation of linear regression is denoted as,

$$\:y=\:\alpha\:x+\:\beta\:$$

where *y* is the dependent, *x* is the independent variable, α the slope, and β the y-intercept. For fitting the linear model, the sum of squared residuals are minimized across all data points. This can be achieved by the ordinary least squares (OLS) method. Since the optimal solution is derived using the OLS method, there was no need or possibility for hyperparameter optimization. In this model, the dependent variable was the hospitalization rate, while the independent variable comprised integers ranging from 1 to 14. Across the 72 windows, we trained the model using data from days 1 to 7 and made predictions for days 8 to 14. In the non-autoregressive scenario, daily wastewater data was included as a second independent variable. To ensure accurate forecasting, all independent variables had to be known, leading us to compensate for the time lag between wastewater and hospitalization data by shifting the wastewater data forward by seven days. This adjustment allowed the fitting window to utilize wastewater data from the previous week and the forecasting window to use the current week’s data. To prevent overfitting, particularly when incorporating the wastewater viral load, we applied ridge regression. Also known as L2 regularization, ridge regression introduces an additional term to the loss function in linear regression, guided by a tunable parameter λ [31]:

$$\sum\,_{i}\!\left(y_i-{\widehat y}_i\right)^{2}+\lambda\sum\,_{j}\beta_{j}^{2}$$

The first part of the loss corresponds to the squared residuals between the observed and predicted values, which corresponds to the normal linear regression loss and the second part corresponds to the additional loss for ridge regression including the regression parameter λ and the sum over the squared coefficients. We utilized the corresponding class in scikit-learn for this purpose. The parameter was fine-tuned on the left-out training data (see Sect. 2.4), within a range of (0, 10].

### ARIMA

Autoregressive Integrated Moving Average (ARIMA) models leverage the statistical properties of stationary data and are widely used for time series forecasting [32]. A stationary time series is characterized by the absence of trends and consistent variation around its mean, allowing for the extraction of short-term random time patterns for forecasting purposes. In this study, we utilized a non-seasonal ARIMA model tailored for short-term periods that are not anticipated to exhibit seasonal effects. The ARIMA models in this context rely on three key parameters:

- p: the number of autoregressive terms.
- d: the degree of differencing applied to achieve stationarity.
- q: the number of lagged forecast errors.

The general forecasting equation for ARIMA is defined as follows:​

$$\begin{aligned}\:\widehat{{y}_{t}}=&\:\mu\:+{\phi\:}_{1}{y}_{t-1}+...+{\phi\:}_{p}{y}_{t-p}\\ &-{\theta\:}_{1}{e}_{t-1}-...-{\theta\:}_{q}{e}_{t-q}\: \end{aligned}$$.

In this equation, ŷ represents the forecast, calculated as the deviation from the mean *µ* of a stationary time series, with φ denoting the slope parameters for the *p* previous values *y*, and *θ* representing the *q* moving average parameters associated with autocorrelation errors *e*. This model learns to predict future values based on the mean of a stationary time series, adjusted for autocorrelation errors and lagged periods. To ensure stationarity, the differencing technique is applied, which involves calculating the differences between consecutive values in the time series [33]. This transformation often leads to stationarity, particularly in first or second order. To identify the optimal parameters (p, d, q), we utilized the auto-ARIMA functionality from the pmdarima library version 1.8.5 [34]. While ARIMA models work by nature autoregressive, it is possible to include exogenous features into the model. In so-called ARIMAX models a term βX is added to the ARIMA equation, where X is the exogenous feature, in our case wastewater viral load, and β is the corresponding coefficient that is estimated by the model [35].

### XGBoost and random forest

Both Random Forest and eXtreme Gradient Boosting (XGBoost) are decision tree-based approaches but differ significantly in their training algorithms. Random Forest creates an unweighted ensemble of decision trees, which is trained in parallel on different subsets of the data using bagging, averaging the predictions [36]. In contrast, XGBoost constructs its decision trees sequentially, correcting the residual errors from the previously trained weighted ensemble using gradient descent [29]. Both models are widely used for tabular data and have also proven effective in time series forecasting [37–42]. Since these models rely on decision trees, they can only extrapolate based on previously observed training data. When predicting values outside the range of the training data, they tend to predict the average or maximum of the observed values. To address this, we applied the differencing technique [33]. That means we calculate from each measured data point to the next the slope of a local tangent. The models are hence tasked to learn and predict the relative change from one datapoint (here day) to another. Before evaluation, the forecast was back-transformed by using the cumulative sum. For Random Forest, we used the scikit-learn library version 1.3.2 RandomForestRegressor class [30], and for XGBoost, we employed the XGBoost library version 1.7.3 [29]. For hyperparameter tuning, we employed a blocked time series cross validation [43] using optuna version 3.2.0 [44] and the same possible hyperparameters (see Table S4) as in our previous study [26].

For both models, we further wanted to test two aspects. First, we wanted to assess whether the inclusion of federal state-level data for training the models would improve the models’ performances in forecasting the hospitalization rates on a country level. Since wastewater data was only available at the country level and by the community, we aggregated daily wastewater data for each federal state by averaging measurements from sewage treatment plants within the state. This data was weighted based on the number of connected citizens, as specified in the dataset for each participating city. This approach aimed to improve prediction accuracy by increasing the dataset’s granularity, which is lost in the aggregation to the country-level. However, only six federal states, Baden-Württemberg, Bayern, Berlin, Hamburg, Nordrhein-Westfalen and Rheinland-Pfalz, had complete and usable datasets for the period under examination. For these six federal states, we used both the regional wastewater viral load and regional hospitalization rates. Secondly, we tested how well these models performed if they were only given the wastewater viral load for forecasting the hospitalization rates, later referred to as cross-modal models.

### Sliding window approach for model training and testing

**Fig. 1:**
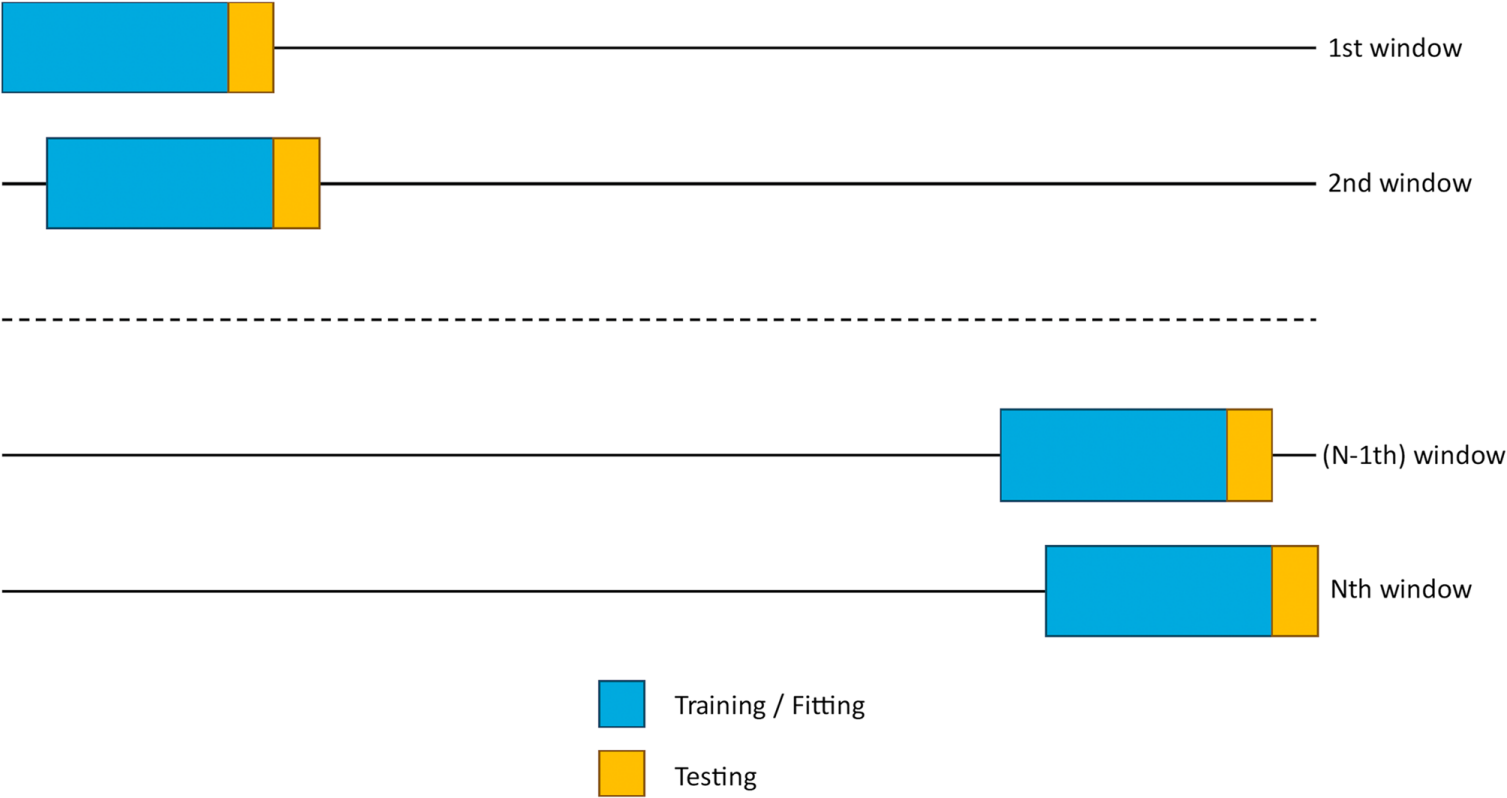
Sliding window approach for retrospective model evaluation. The time series was split into N training and testing windows. Models were trained on the training/fitting windows (blue) and then forecasted 7 days ahead. Predictions were compared against real values observed in the testing window (yellow)

To account for potential changes in conditions during the study periods - such as new safety regulations, vaccines, or virus variants - and to provide more test windows for a more reliable evaluation of the model’s performance, we employed a sliding window approach for both training and testing data (Fig. 1).

In this approach, we predict the hospitalization rate for a seven-day period (prediction window) based on a preceding set of data points (context window). The size of the prediction window is set to seven days because of the time lag between wastewater viral load and hospitalization rate observed via cross-correlation analysis of less than two weeks (see Results section). Additionally, a prediction window of seven days corresponds to the weekly cycle of usual data reporting (daily to weekly. After each prediction, both windows are shifted forward by seven days, ensuring no overlap between successive prediction windows. Applying this method throughout the entire period for which both wastewater and hospitalization data are available results in 68 testing windows.

This approach, which is similar to one used in our previous study [26], simulates a scenario where the models are trained on past data, assuming data completeness up to each point in time.

**Fig. 2:**
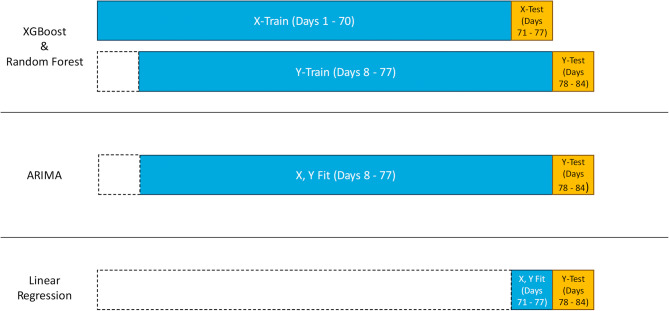
Model training and testing. For XGBoost and Random Forest the time series is shifted by 7 days to obtain a target vector to learn the forecasting task. Here a context length of 70 days (blue) is used. ARIMA learns this representation internally is therefore just assigned to a 70 day context window (blue). Linear regression can oversmooth large context windows. Therefore, a 7 day context window (blue) is assigned. The windows are offset accordingly, such that all models predict the same 7 days (yellow)

Given that the models operate in fundamentally different ways, we assigned them context windows of varying sizes (Fig. 2). For tree-based models like XGBoost and Random Forest, which benefit from larger context windows, we used a window size of 70 days to be consistent with a previously published study [26]. Additionally, for model training, we shifted the time series by 7 days to obtain the corresponding target vector. Similarly, ARIMA, which identifies patterns in the data, also requires a larger sample size, so it was assigned a 70-day context window as well. Here shifting the data is not necessary as ARIMA employs a different learning strategy (see methods). While linear regression generally improves with more data, using a large context window can smooth out smaller trends. In fact, using a 70-day window for linear regression in this case could be counterproductive, as it may obscure short-term trends relevant to the seven-day forecast. Instead, we opted to fit the linear regression using only the last seven days of training data to better capture recent changes. For a valid comparison between the models, it is essential that they forecast the same days and have the same number of forecasting windows. To achieve this, we introduced offsets at the beginning of the time series, ensuring that the initial test forecasting windows are aligned across all three models.

### Model evaluation and comparison

To assess the performance of the models on the testing windows, we used the mean absolute error (MAE) [45] and the mean absolute percentage error (MAPE) [46] as metrics:

$$\begin{aligned}&\:MAE\:=\frac{1}{n}{\sum\:}_{i=1}^{n}\:\left|{Y}_{i}-{\widehat{Y}}_{i}\right|\:\:;\\ &MAPE\:=\frac{1}{n}{\sum\:}_{i=1}^{n}\left|\frac{{Y}_{i}-{\widehat{Y}}_{i}}{{Y}_{i}}\right|*100\: \end{aligned}$$

Where Y represents the observed values, Ŷ represents the predicted values, and n is the number of data points, which in our case corresponds to the length of the testing window (7 days). The MAPE expresses the deviation of the prediction from the observed data as a percentage, making it a more intuitive measure compared to the MAE. However, due to this normalization, the MAPE is high for deviations in small scales, e.g. 50% if the predicted value is 1 but the observed value is 2 and can also be problematic when Y approaches 0. Therefore, we decided to record both the MAE and the MAPE. Both MAE and MAPE have been commonly used for assessing predictive modeling performances in COVID-19 forecasting [47–50].

In order to test for statistical differences between two models, we declare them to be statistically different if the 95% confidence intervals of their mean MAPE does not overlap.

A limitation of both MAE and MAPE is that they are only applicable to point forecasts and do not consider the uncertainty of the forecast. There are several measures for probabilistic forecasts that can assess the quality of the uncertainty quantification. One of them is the interval score (IS) [51] which has also been used in the context of pandemic forecasting [52, 53]. The interval score combines the width of the prediction interval with penalizing uncovered true values (coverage). A coverage of 100% means that all true values fall into the prediction interval. The optimal coverage therefore depends on the chosen nominal level, for instance with a 95% confidence level, optimal coverage would be 95%. The interval score is defined as,

$$\:{\text{IS}}_{\alpha\:}\left(l,u;y\right)=\left(u-l\right)+\frac{2}{\alpha\:}\text{(l-y}{)\delta\:}_{l}\text{+}\frac{2}{\alpha\:}\text{(y-u}{)\delta\:}_{u}$$

with

$$\:{\delta\:}_{l}\left\{\begin{array}{c}1\:\text{i}\text{f}\text{}y<l\\\:0\:\text{e}\text{l}\text{s}\text{e}\end{array}\right.\:;\:{\delta\:}_{u}\left\{\begin{array}{c}1\:\text{i}\text{f}\text{}y>u\\\:0\:\text{e}\text{l}\text{s}\text{e}\end{array}\right.$$

Where *y* is the true value, l is the lower limit of the prediction interval, *u* is the upper limit of the prediction interval, and α the significance level. The first term corresponds to the sharpness and the second term penalizes small coverage. Since the interval score is scale dependent – larger true values lead to larger interval scores, we further decided to normalize the IS by dividing it by the true value *y*. Since it is common practice to report the 95% prediction intervals, we decided to calculate the interval score for α = 0.05. Additionally, we will also report the relative sharpness (width of prediction interval divided by true value). The coverage is independent of the scale.

All models were evaluated on the original scale (not log-transformed).

## Results

### Wastewater viral load as an early warning indicator

**Fig. 3:**
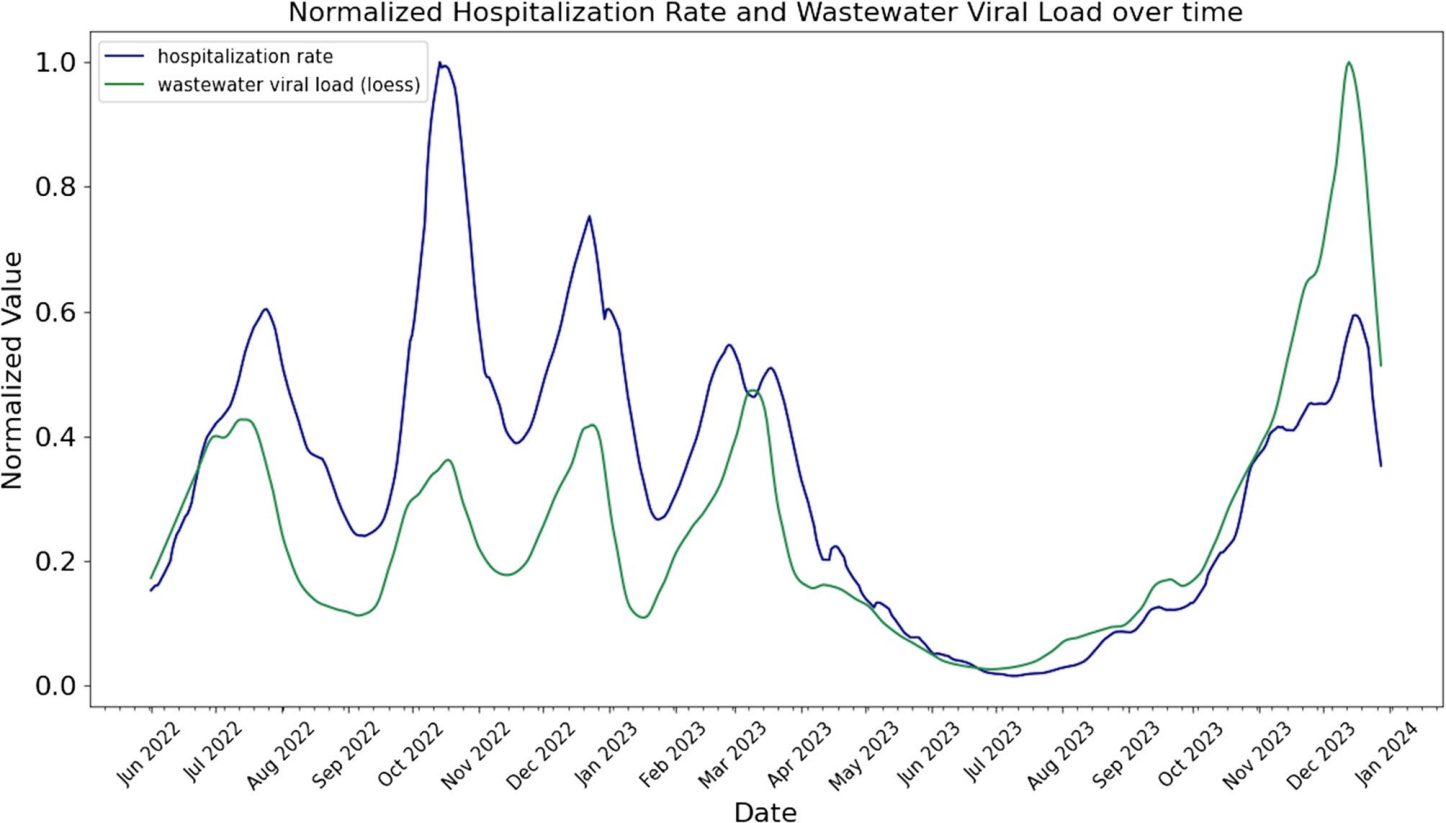
Normalized time series – smoothed hospitalization and daily-interpolated wastewater viral load from July 2022 to January 2024 in Germany. Prior to plotting, the data were normalized using min-max normalization. Hospitalization is plotted in blue, wastewater viral load in green

**Fig. 4:**
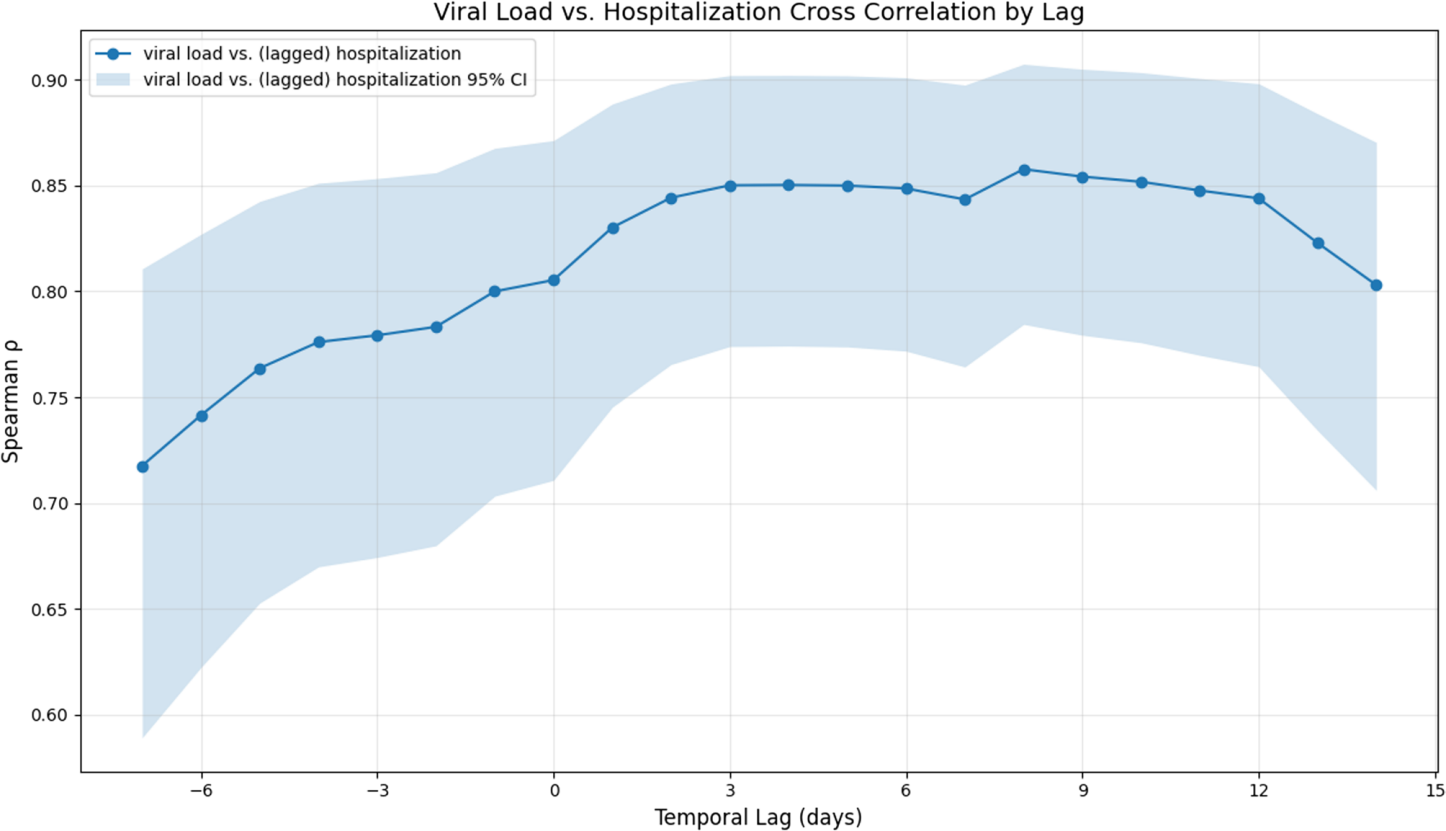
Spearman correlation coefficient between raw daily hospitalization rate and raw wastewater viral load for different time shifts (lag values) on national level.

In Fig. 3 we plotted the normalized and smoothed hospitalization rates and the LOESS interpolated and normalized daily wastewater viral load for the time period of July 2022 to January 2024. It can be seen that the time series show exponential behavior and follow the same up- and down-trends. The plot also reveals that the relation between the scales of both time series for hospitalization and wastewater viral load changed throughout the observed period. While hospitalization showed its maximal peak at the end of September 2022, wastewater viral load peaked most in December 2023. Next, we calculated the correlation coefficients between the raw daily hospitalization rates and raw, weekly wastewater viral load. The results can be seen in Table 1. Spearman’s analysis suggests a high correlation of around 81%. Additionally, we wanted to derive the time lag with the highest correlation, i.e. the number of days the time series need to be shifted against each other to achieve maximal correlation. In Fig. 4 we also show how Spearman correlation coefficient changes with different number of days shifted. Here it can be seen that a negative lag, i.e. shifting the hospitalization more toward the future, drastically reduces the correlation while a positive lag increases the correlation up to its maximum at 8 days and then slowly decreases towards 14 days. Even though not necessarily statistically significant (the confidence intervals overlap), a clear trend can be observed. Additionally, we calculated the correlation between the smoothed hospitalization rate and the interpolated daily wastewater viral load as well as between the smoothed hospitalization rate and the raw wastewater viral load (see Figure S1). Here the maximal correlation was reached after 10 days. Altogether our analysis shows that wastewater viral load data has impeding trends.

**Table 1:** Spearman correlation between Raw daily hospitalization rate (by 8 days shifted daily hospitalization rate) and Raw wastewater viral load, and 95% confidence intervals. All *p*-values were below 1 × 10^−10^

	Viral Load
Hospitalization	0.81; (0.71, 0.87)
Shifted Hospitalization	0.86; (0.78, 0.90)

### Limited benefit of wastewater viral load for forecasting of hospitalization

**Fig. 5:**
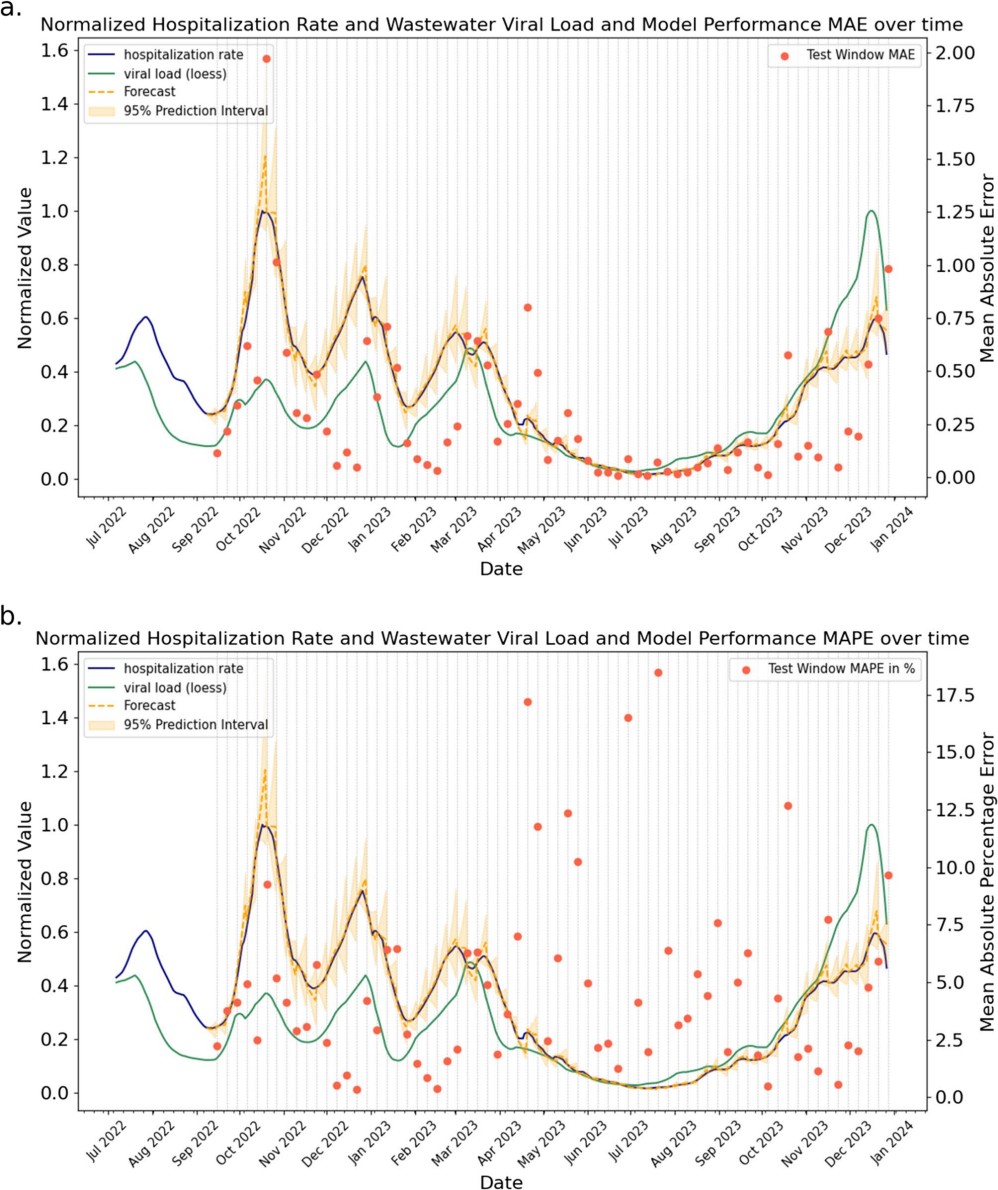
ARIMA performance including wastewater viral load on test windows on national level. Both hospitalization rate (blue) and wastewater viral load (green) as well as the corresponding forecast (orange lines) and 95% prediction interval (yellow) are plotted normalized. Along with this the mean absolute errors (**a**) and mean absolute percentage errors (**b**) per test window are displayed (orange dots)

The model performances on forecasting the hospitalization 7 days ahead are displayed in Table 2. The results are given as the mean MAE and mean MAPE over the 68 testing windows including the corresponding 95% prediction interval. All models were tested both autoregressive and with the inclusion of the wastewater viral load. Altogether the ARIMA model turned out to perform significantly better than all tested alternatives, apart from the Random Forest model with wastewater data, according to the confidence intervals of the MAPE, with small overlaps in the confidence intervals of the MAE. The inclusion of wastewater viral load as an exogenous predictor did not improve prediction performance, according to the largely overlapping confidence intervals of the models with and without the inclusion of wastewater viral load data of MAPE and MAE. Notably, the tree-based models showed a somewhat reduced mean forecasting error and interval width, but no significant overall effect. This is also supported by the probabilistic forecast metrics, sharpness, coverage, and interval score (see Table 3). ARIMA without and with (ARIMAX) the inclusion of wastewater viral load data show similar coverage (94% vs. 92%) and relative sharpness (24.3 vs. 25.7). However, we can see a significant difference in the relative interval score of the prediction, where the ARIMA model has a lower mean value than the ARIMAX model (39 vs. 47). Figure 5 shows the model forecasts and performances over time on the example of the ARIMA model.

**Table 2:** Model performances on forecasting hospitalization rates 7 days ahead in terms of point forecast metrics. Models were evaluated on 68 testing windows using the MAE and MAPE. Here the mean MAE and mean MAPE are displayed with the corresponding 95% confidence interval. Models were trained and tested autoregressive (AR) yes or no

Model	AR	MAE	MAPE in %
Linear Regression	X	0.54; (0.37, 0.70)	8.47; (7.07, 9.88)
		0.58; (0.33, 0.83)	8.50; (6.43, 10.56)
Ridge Regression	X	0.52; (0.38, 0.66)	8.05; (6.77, 9.34)
		0.52; (0.38, 0.65)	8.05; (6.77, 9.34)
ARIMA	X	0.29; (0.21, 0.37)	4.76; (3.79, 5.72)
		0.30; (0.21, 0.38)	5.42; (4.15, 6.70)
Random Forest	X	0.65; (0.38, 0.92)	8.54; (7.00, 10.08)
		0.44; (0.28, 0.61)	6.62; (5.29, 7.95)
XGBoost	X	0.66; (0.41, 0.90)	8.68; (7.09, 10.27)
		0.48; (0.32, 0.64)	7.33; (6.01, 8.65)

**Table 3:** ARIMA (purely autoregressive) and ARIMAX (including wastewater data as exogenous variable) performances on forecasting hospitalization rates 7 days ahead in terms of probabilistic forecast metrics. Model were evaluated on 68 testing windows using the relative sharpness, coverage and relative integrated score (IS) calculated on the 95% confidence interval. Mean values are displayed with corresponding 95% confidence intervals

Model	Relative Sharpness	Coverage	Relative IS (95% CI)
ARIMA	24.32 (22.85, 25.79)	94 (89, 99)	39.43 (32.75, 47.10)
ARIMAX	25.66 (24.01, 27.30)	92 (85, 95)	47.30 (36.97, 57.63)

For Random Forest and XGBoost, we further tested whether the inclusion of the federal-state wastewater viral load and hospitalization rates could improve the model performances. Here we only found a mean MAPE of 11.06% for Random Forest and of 11.30% for XGBoost (Table 4), thus not improving the models that were solely trained on country-level data.

**Table 4:** Decision tree-based model performances on forecasting hospitalization rates 7 days ahead including regional wastewater viral load and hospitalization data. Models were evaluated on 68 testing windows using the MAE and MAPE. Here the mean MAE and mean MAPE are displayed with the corresponding 95% confidence interval. Models were trained including regional wastewater viral load data as well as regional hospitalization rates from 6 German federal States

Model	MAE	MAPE in %
Random Forest - Regional	0.74; (0.49, 0.98)	11.06; (9.46, 12.67)
XGBoost - Regional	0.75; (0.54, 0.96)	11.30; (9.94, 12.66)

Finally, we also built cross-modal Random Forest and XGBoost models, where the hospitalization was forecasted only based on the wastewater viral load as a predictor. Here we found a Random Forest performance of 6.66% and XGBoost performance of 7.23% mean MAPE (see Table 5). Comparing these results with the results from the previous part we observe overlapping confidence intervals: For Random Forest (5.29, 7.95) with both wastewater viral load and hospitalization compared to (5.33, 8.00) cross-modal (without hospitalization data as predictor) and for XGBoost (6.01, 8.65) versus (5.85, 8.62), respectively. From this, we can derive that both models can perform as well as the models that were either trained purely autoregressive or also including wastewater viral load data.

**Table 5:** Decision tree based cross-modal model performances on forecasting hospitalization rates 7 days ahead. Models were evaluated on 68 testing windows using the MAE and MAPE. Here the mean MAE and mean MAPE are displayed in percent with the corresponding 95% confidence interval. Models were trained cross-modal, i.e. Wastewater viral load was used as sole predictor for hospitalization rates

Model	MAE	MAPE in %
Random Forest - Cross-Modal	0.44; (0.28, 0.61)	6.66; (5.33, 8.00)
XGBoost - Cross-Modal	0.49; (0.31, 0.66)	7.23; (5.85, 8.62)

## Discussion

The COVID-19 pandemic underlined the need for reliable measures and tools to effectively monitor and forecast the spread of the disease. In this study, we evaluated the wastewater viral load data collected by the AMELAG project in Germany, examining its correlation with COVID-19 hospitalization and incorporating it as a predictor in otherwise autoregressive models to forecast hospitalization rates 7 days ahead.

The observed changes over time in the relationship between hospitalization and wastewater viral load -with each time series peaking at different stages (see Fig. 3)- suggest that factors such as variations in wastewater testing protocols, differences in hospital reporting practices, and a possible decline in the reporting of all hospitalized patients following the downgrade of COVID-19 from a Public Health Emergency of International Concern in early 2023 may have influenced the data. Additionally, the overtime-changing variant of the pathogen affects the viral load [54], as well as herd immunity, or less severe infections may have changed the relationship between viral load measures in wastewater and hospitalization. Despite these fluctuations, the high Spearman correlation (above 0.8) indicates a strong monotonic relationship. This is expected since both are causes of infections of COVID-19 in the population, first leading to signals in wastewater and later to potential severe progression with subsequent hospitalization.

The cross-correlation analysis showed a high Spearman correlation (around 0.8). The lag analysis further confirmed that wastewater viral load precedes hospitalization rate by up to 8 days, with an uncertainty of 2 days, due to the sampling procedure, indicating a potential lead time advantage in using wastewater data for early warning of increasing hospitalization. Noteworthy, the maximum lag found is based on the central estimates of the cross correlation. But, the broad, overlapping confidence intervals and the flatness of the curve mean the true lead could also be less or more. The uncertainty is substantial, so the precise lead time should not be over-interpreted. Using the smoothed time series returned lead times to even 10 days, which however can come from the left-tailed moving average which biases the time series to the past. Here, the temporal smoothing introduced via LOESS acts as a noise filter, which may lead to a certain inflation of the correlation between wastewater viral load and hospitalization rate [22].

In the literature, the lead time between wastewater viral load and hospitalization varies largely. Peng et al. found that the time lag depends on the variant with delta with up to 15 days and omicron with 7–10 days in Canada [55]. Hill et al. found a lead time of 10 days in the state of New York [56], while Schenk et al. found lead times of 2–12 days across 40 US states arguing that larger wastewater systems with more than 70,000 people connected showed tighter alignment of 2–7 days lead [57]. Considering that our time frame has been mostly dominated by the omicron variant and its subvariants, the lead time identified in our studies matches the results of the literature.

The observed time lag between wastewater viral load and hospitalization rates motivated us to evaluate, whether forecasting models could benefit from including wastewater surveillance as an exogenous predictor. However, our analysis could not show any significant improvement. In general, ARIMA emerged as the overall best method for short-term forecasting, which is in alignment with findings in our previous study [26]. The model performance over time (Fig. 5) agrees with the model performances in the literature. Without additional covariates and careful calibration, these forecasting models typically cannot predict the turning points of waves but help to accurately forecast in periods of rather stable increases or decreases of case counts [2]. At the same time, including wastewater viral load as an exogenous predictor did not significantly enhance forecasting performance. This was confirmed by both the point forecast metrics MAE and MAPE, as well as the probabilistic forecast metrics coverage and relative interval score. An explanation for the non-present improvement of models with wastewater viral load data is that both data modalities, wastewater viral load and hospitalization rates, have a high level of redundancy relative to each other, as reflected by their strong correlation. Also, the dynamically changing number of participating treatment plants could have led to a difficulty in learning the representation of the wastewater data. Additionally, the inclusion of wastewater data can lead to overfitting. This could be the reason that only the tree-based models showed tendencies to improve with wastewater viral load data, since they were additionally tuned via cross validation. Note that a COVID-19 infection is the common cause behind both data modalities. The observed strong correlation is likely the reason why our cross-modal models performed similarly well as the autoregressive ones only using the hospitalization rate. The utility of cross-modal models depends critically on real-time data availability. Our analysis assumes synchronized hospitalization and wastewater data, but this may not reflect operational realities. If hospitalization data lacks timeliness, wastewater-based models offer a viable predictive alternative due to simpler aggregation (national data from less than 200 treatment plants vs. numerous hospitals). While resource requirements for data collection remain unquantified here, these models gain value when applied to case forecasting (including asymptomatic infections). Establishing a wastewater-to-case relationship could eventually reduce dependence on individual-level testing.

Models using regional wastewater viral load data did not outperform those based on aggregated country-level data, despite the expectation that more granular data would provide additional insights. One possible explanation is that wastewater sampling is conducted at distinct treatment plants, which are regionally not necessarily distributed in the same way as the population. Furthermore, models for regional data may fail to account for a varying population density within each catchment area. For example, a treatment plant may serve both urban and rural zones. This location-population misalignment may lead to discrepancies in the time lag between viral load peaks and according hospitalization rates. In contrast, country-level data tend to be smoother, reducing the impact of such differences between regions.

In their study on wastewater surveillance [58], Shah et al. pointed out that wastewater monitoring can identify the presence of SARS-CoV-2 even before any clinical cases are reported. They further underscore the potential of wastewater-based epidemiology to deliver prompt insights for public health decision-making. These observations were corroborated by German studies [10, 12, 13]. Hill et al. [56] discovered that incorporating wastewater data into a multivariate model for predicting hospitalization across 56 counties in New York State enhanced the model’s performance by 15%. Similarly, Li et al. [59] demonstrated that wastewater data could effectively forecast hospitalization in the USA using a Random Forest approach. On the other hand, Pilz et al. [16] showed that while wastewater data qualitatively predicted hospitalization waves, it was inadequate for quantitatively predicting prevalence on the city-level. Their study relied on wastewater viral load data from a single German federal state. Although our study did not observe significant enhancements from including wastewater viral load, our findings are consistent with the literature and emphasize the potential of wastewater viral load as an early detection indicator and as a sole predictor for cross-modal forecasting.

Of course, our study has limitations. Our analysis assumes wastewater and hospitalization data are available exactly on the dates recorded. In practice, there can be delays in data collection, processing, and reporting, which are difficult to quantify and may vary over time and by location. The RKI states [60]: “The time delay from sampling to transmission to the RKI and publication in the infection radar can be several weeks; for most sewage treatment plants it is less than a week.” As a result, our findings reflect an ideal scenario of real-time data availability, which may not be achievable in operational settings. This introduces some uncertainty regarding the practical applicability of our results and in the time lag found. Additionally, we used interpolated and smoothed data in our predictive models, which may introduce a slight over-optimism.

Our results suggest that systematic monitoring of wastewater could provide a benefit in terms of early detection of later hospitalization trends. Additionally, our focus was on short-term, seven-day forecasts. While longer-term forecasting and scenario planning are crucial during pandemics, short-term forecasts can still offer valuable insights, such as in the allocation of hospital resources or the use of protective measures to reduce viral spread.

## Conclusion

The COVID-19 pandemic highlighted the need for reliable monitoring tools, with wastewater viral load emerging as a promising early indicator of outbreaks. This study evaluated wastewater data from the AMELAG project in Germany, assessing its correlation with COVID-19 hospitalization and its potential as a predictor in forecasting models. Although strong correlations were found, particularly with a time lag of up to 8 days, incorporating wastewater viral load into predictive models did not significantly improve forecasting accuracy of hospitalization rates. On the other hand, using wastewater viral load as a sole predictor of hospitalization rates demonstrated a prediction performance comparable to using hospitalization rates alone, as shown by the overlapping confidence intervals of the performance metrics. This suggests that wastewater data is valuable as an early indicator as well as a predictor of epidemiological surveillance, especially as it provides information on the community level. At the same time, a combination of wastewater information with hospitalization rates provides limited benefit.

Models using regional wastewater data did not outperform those based on national data, likely due to discrepancies between the regional distribution of wastewater plants and population density. Altogether, despite limitations, including data interpolation and a focus on short-term forecasts, our study reinforces the potential of wastewater surveillance as an early warning tool and predictor of epidemiological parameters. Future research should explore its broader application for other pathogens, potentially in combination with other data modalities such as social media symptom reporting [61] as well as the real-time applicability.

## Supplementary Information

Supplementary Material 1.

Supplementary Material 2.

## Data Availability

The hospitalization rates and the wastewater viral load data can be found on GitHub: https://github.com/robert-koch-institut. These datasets are publicly available. The authors are not the owners of the data and cannot redistribute them directly. Please refer to the original source for access and licensing terms. The code is available at: https://github.com/SCAI-BIO/wasterwater_analysis.
