# Supplementary material for: Wastewater as an early indicator for short-term forecasting COVID-19 hospitalization in Germany
Source: BMC Public Health. 2025 Aug 25;25:2910. doi: 10.1186/s12889-025-24149-2 (PMC12376350; doi:10.1186/s12889-025-24149-2)
Supplement: Supplementary file 1 — Supplementary Material 1. [file 12889_2025_24149_MOESM1_ESM.docx]

Supplementary Material

| Model | Hyperparameter | Search Space | Sampler |
| --- | --- | --- | --- |
| Random Forest | Min_samples_split Min_samples_leaf | [2,4,8,16] [1,2,4] | Grid-Search |
| XGBoost | Learning rate Max_depth  Reg_lambda Reg_alpha | (0.01,0.5) (2,12) (0,10) (0,10) | TPE |

**Table S1: Hyperparameters for Random Forest and XGBoost models** The brackets correspond to discrete values, while the parentheses correspond to continuous parameter ranges. Hyperparameters were either optimized by using a grid-search or with a tree-structured Parzen estimator from optuna. As the model’s hyperparameters were tuned for each context window individually we did not provide the final choices.
