# Supplementary material for: Wastewater as an early indicator for short-term forecasting COVID-19 hospitalization in Germany
Source: BMC Public Health. 2025 Aug 25;25:2910. doi: 10.1186/s12889-025-24149-2 (PMC12376350; doi:10.1186/s12889-025-24149-2)
Supplement: Supplementary file 2 — Supplementary Material 2. [file 12889_2025_24149_MOESM2_ESM.docx]

Supplementary Material


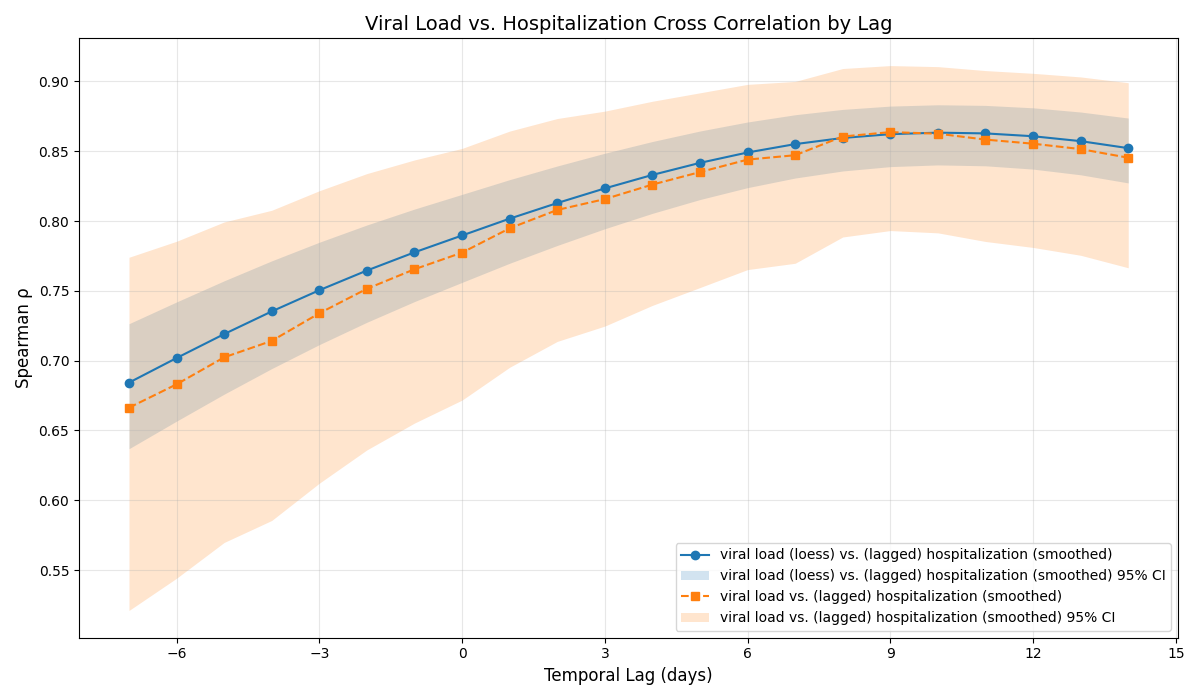


Figure S1: **Spearman correlation coefficient between smoothed daily hospitalization rate and raw wastewater viral load (orange) and interpolated daily wastewater viral load (blue) for different time shifts (lag values) on national level.**

| Location | Federal State | Population | Time Frames |
| --- | --- | --- | --- |
| Aachen | NW | 206424 | 22.2022-35.2022 ; 36.2022-51.2022 ; 01.2023-52.2023 |
| Aalen | BW | 30000 | 48.2023-52.2023 |
| Altötting | BY | 34550 | 22.2022-23.2022 ; 25.2022-25.2022 ; 28.2022-42.2023 ; 43.2023-43.2023 ; 45.2023-45.2023 ; 47.2023-50.2023 ; 51.2023-52.2023 |
| Andernach | RP | 47086 | 40.2022-52.2023 |
| Aschaffenburg | BY | 102806 | 41.2023-43.2023 ; 45.2023-45.2023 ; 48.2023-51.2023 |
| Augsburg | BY | 380000 | 22.2022-52.2022 ; 02.2023-14.2023 ; 15.2023-20.2023 ; 21.2023-27.2023 ; 29.2023-43.2023 ; 45.2023-52.2023 |
| Bad Kreuznach | RP | 75000 | 40.2022-08.2023 ; 09.2023-52.2023 |
| Bad Mergentheim | BW | 27257 | 45.2023-52.2023 |
| Bad Reichenhall | BY | 19480 | 45.2023-52.2023 |
| Bayreuth | BY | 88906 | 42.2022-17.2023 ; 18.2023-43.2023 ; 45.2023-52.2023 |
| Berchtesgaden | BY | 25000 | 45.2023-52.2023 |
| Berg | BW | 14000 | 45.2023-52.2023 |
| Berlin | BE | 1500000 | 22.2022-26.2022 ; 27.2022-41.2023 ; 42.2023-52.2023 |
| Bernburg | ST | 37000 | 45.2022-50.2022 ; 01.2023-02.2023 ; 03.2023-10.2023 ; 12.2023-19.2023 ; 21.2023-51.2023 ; 52.2023-52.2023 |
| Bonn | NW | 183361 | 22.2022-51.2022 ; 01.2023-51.2023 |
| Bottrop | NW | 732616 | 22.2022-51.2022 ; 01.2023-51.2023 |
| Brandenburg an der Havel | BB | 82881 | 45.2023-51.2023 |
| Braunschweig | NI | 280000 | 45.2023-52.2023 |
| Bremen | HB | 595000 | 22.2022-25.2022 ; 26.2022-26.2022 ; 28.2022-19.2023 ; 21.2023-52.2023 |
| Büdingen | HE | 14462 | 45.2023-50.2023 |
| Celle | NI | 71244 | 45.2023-52.2023 |
| Cottbus | BB | 150000 | 45.2023-51.2023 |
| Dessau | ST | 83000 | 44.2022-49.2022 ; 01.2023-02.2023 ; 03.2023-05.2023 ; 07.2023-09.2023 ; 11.2023-18.2023 ; 20.2023-29.2023 ; 31.2023-50.2023 ; 52.2023-52.2023 |
| Dinslaken | NW | 56812 | 22.2022-51.2022 ; 01.2023-51.2023 |
| Donaueschingen | BW | 100000 | 45.2023-52.2023 |
| Dortmund-Deusen | NW | 399425 | 22.2022-51.2022 ; 01.2023-51.2023 |
| Dortmund-Scharnhorst | NW | 113439 | 22.2022-51.2022 ; 01.2023-51.2023 |
| Dresden | SN | 700000 | 22.2022-51.2022 ; 01.2023-50.2023 |
| Duisburg | NW | 242172 | 22.2022-51.2023 |
| Döbeln | SN | 21092 | 12.2023-52.2023 |
| Düsseldorf (Nord) | NW | 333122 | 44.2023-50.2023 |
| Düsseldorf (Süd) | NW | 340577 | 44.2023-50.2023 |
| Ebersberg | BY | 12500 | 45.2023-52.2023 |
| Emschermündung | NW | 906222 | 22.2022-51.2022 ; 01.2023-51.2023 |
| Eriskirch | BW | 36000 | 45.2023-52.2023 |
| Erlangen | BY | 170332 | 49.2022-51.2022 ; 01.2023-52.2023 |
| Eschweiler | NW | 68244 | 22.2022-51.2022 ; 01.2023-52.2023 |
| Flensburg | SH | 115000 | 45.2023-52.2023 |
| Frankfurt | BB | 65444 | 45.2023-51.2023 |
| Frankfurt-Niederrad | HE | 903368 | 45.2023-49.2023 |
| Frankfurt-Sindlingen | HE | 245495 | 45.2023-49.2023 |
| Freilassing | BY | 18500 | 33.2023-33.2023 ; 35.2023-43.2023 ; 45.2023-51.2023 |
| Freising | BY | 51617 | 41.2023-52.2023 |
| Fulda | HE | 95000 | 47.2023-47.2023 ; 49.2023-51.2023 |
| Germersheim | RP | 28318 | 41.2022-43.2023 ; 44.2023-44.2023 ; 46.2023-52.2023 |
| Gerwisch | ST | 278000 | 45.2022-50.2022 ; 01.2023-02.2023 ; 03.2023-10.2023 ; 11.2023-18.2023 ; 20.2023-29.2023 ; 31.2023-50.2023 ; 52.2023-52.2023 |
| Glonn | BY | 5300 | 22.2022-22.2022 ; 24.2022-33.2022 ; 35.2022-46.2022 ; 48.2022-48.2022 ; 50.2022-43.2023 ; 45.2023-45.2023 ; 46.2023-52.2023 |
| Grafing | BY | 14900 | 22.2022-47.2022 ; 49.2022-52.2022 ; 02.2023-45.2023 ; 46.2023-52.2023 |
| Greifswald | MV | 61963 | 45.2023-51.2023 |
| Grimma | SN | 19800 | 12.2023-52.2023 |
| Göppingen | BW | 300000 | 48.2023-52.2023 |
| Görlitz | SN | 100000 | 41.2023-52.2023 |
| Göttingen | NI | 170000 | 45.2023-52.2023 |
| Halberstadt | ST | 35345 | 02.2023-10.2023 ; 12.2023-19.2023 ; 21.2023-51.2023 ; 52.2023-52.2023 |
| Halle (Saale) | ST | 337000 | 01.2023-05.2023 ; 07.2023-10.2023 ; 12.2023-18.2023 ; 21.2023-24.2023 ; 26.2023-42.2023 ; 43.2023-47.2023 ; 48.2023-51.2023 |
| Hamburg 01 | HH | 581423 | 22.2022-52.2023 |
| Hamburg 02 | HH | 1464784 | 22.2022-52.2023 |
| Hanau | HE | 140000 | 47.2023-51.2023 |
| Hannover-Gümmerwald | NI | 437416 | 47.2023-52.2023 |
| Hannover-Herrenhausen | NI | 303144 | 47.2023-52.2023 |
| Heidelberg | BW | 161477 | 22.2022-23.2023 ; 24.2023-52.2023 |
| Hetlingen | SH | 540000 | 45.2023-52.2023 |
| Hildesheim | NI | 104230 | 45.2023-52.2023 |
| Hof | BY | 100000 | 22.2022-31.2022 ; 34.2022-52.2023 |
| Husum | SH | 40000 | 45.2023-52.2023 |
| Ingolstadt | BY | 177591 | 39.2023-51.2023 ; 52.2023-52.2023 |
| Jena | TH | 116186 | 22.2022-24.2022 ; 25.2022-31.2022 ; 33.2022-36.2022 ; 38.2022-13.2023 ; 15.2023-16.2023 ; 18.2023-27.2023 ; 29.2023-32.2023 ; 34.2023-49.2023 |
| Kaiserslautern | RP | 140000 | 40.2022-16.2023 ; 18.2023-51.2023 |
| Kassel | HE | 271330 | 45.2023-46.2023 ; 49.2023-51.2023 |
| Kellinghusen | SH | 8282 | 45.2023-52.2023 |
| Kiel | SH | 351644 | 45.2023-52.2023 |
| Koblenz | RP | 107200 | 40.2022-52.2023 |
| Köln | NW | 863455 | 22.2022-23.2022 ; 25.2022-07.2023 ; 08.2023-14.2023 ; 15.2023-19.2023 ; 21.2023-23.2023 ; 24.2023-24.2023 ; 26.2023-47.2023 ; 49.2023-50.2023 |
| Königsbach | BW | 27239 | 27.2023-41.2023 ; 45.2023-52.2023 |
| Königsbrunn | BY | 28100 | 22.2023-23.2023 ; 24.2023-37.2023 ; 38.2023-39.2023 ; 40.2023-45.2023 ; 47.2023-48.2023 ; 50.2023-52.2023 |
| Köthen | ST | 36000 | 45.2022-48.2022 ; 50.2022-50.2022 ; 01.2023-02.2023 ; 03.2023-10.2023 ; 12.2023-19.2023 ; 21.2023-51.2023 ; 52.2023-52.2023 |
| Landau in der Pfalz | RP | 54708 | 40.2022-52.2023 |
| Leonberg | BW | 80000 | 48.2023-52.2023 |
| Ludwigshafen (BASF) | RP | 250000 | 42.2022-51.2023 |
| Lübeck | SH | 245000 | 45.2023-52.2023 |
| Mainz | RP | 250000 | 40.2022-11.2023 ; 12.2023-52.2023 |
| Marburg | HE | 77933 | 45.2023-51.2023 |
| Montabaur | RP | 24900 | 40.2022-52.2022 ; 03.2023-52.2023 |
| Mönchengladbach | NW | 411656 | 22.2022-51.2022 ; 01.2023-52.2023 |
| Mühlacker-Lomersheim | BW | 25311 | 28.2023-41.2023 ; 45.2023-52.2023 |
| München | BY | 1240000 | 22.2022-24.2022 ; 25.2022-49.2022 ; 50.2022-32.2023 ; 33.2023-37.2023 ; 38.2023-51.2023 |
| Naumburg | ST | 40000 | 45.2022-50.2022 ; 01.2023-03.2023 ; 06.2023-06.2023 ; 08.2023-10.2023 ; 12.2023-19.2023 ; 21.2023-51.2023 |
| Neu-Ulm | BY | 220000 | 32.2023-52.2023 |
| Neubrandenburg | MV | 64003 | 45.2023-51.2023 |
| Neustadt an der Weinstraße | RP | 53000 | 22.2022-27.2023 ; 30.2023-52.2023 |
| Nürnberg | BY | 588807 | 51.2022-51.2023 ; 52.2023-52.2023 |
| Offenburg | BW | 120000 | 45.2023-52.2023 |
| Oldenburg | NI | 170000 | 45.2023-52.2023 |
| Osnabrück | NI | 156679 | 45.2023-52.2023 |
| Passau | BY | 54000 | 46.2022-51.2022 ; 02.2023-10.2023 ; 13.2023-13.2023 ; 15.2023-18.2023 ; 21.2023-43.2023 ; 45.2023-52.2023 |
| Pforzheim | BW | 125000 | 48.2023-52.2023 |
| Piding | BY | 9070 | 22.2022-51.2022 ; 52.2022-32.2023 ; 34.2023-43.2023 ; 45.2023-52.2023 |
| Pirmasens-Blümelstal | RP | 60000 | 40.2022-47.2022 ; 48.2022-51.2022 ; 01.2023-22.2023 ; 24.2023-52.2023 |
| Potsdam | BB | 78000 | 22.2023-38.2023 ; 39.2023-51.2023 |
| Primasens-Felsalbe | RP | 15300 | 40.2022-40.2022 ; 41.2022-47.2022 ; 48.2022-51.2022 ; 02.2023-22.2023 ; 24.2023-52.2023 |
| Ratzeburg | SH | 14894 | 47.2023-52.2023 |
| Regensburg | BY | 279474 | 11.2023-14.2023 ; 16.2023-25.2023 ; 27.2023-43.2023 ; 45.2023-45.2023 ; 47.2023-52.2023 |
| Rollsdorf | ST | 40867 | 34.2022-09.2023 ; 12.2023-52.2023 |
| Rostock | MV | 240420 | 19.2023-51.2023 |
| Saarbrücken | SL | 123994 | 23.2022-42.2023 ; 45.2023-52.2023 |
| Saarlouis | SL | 43027 | 45.2023-52.2023 |
| Schleswig | SH | 49743 | 49.2023-52.2023 |
| Schwabmünchen | BY | 18000 | 39.2023-52.2023 |
| Schweinfurt | BY | 82500 | 22.2022-52.2023 |
| Schwerin | MV | 129460 | 46.2023-51.2023 |
| Schwäbisch Hall | BW | 100000 | 48.2023-52.2023 |
| Schönebeck | ST | 72000 | 30.2023-31.2023 ; 33.2023-36.2023 ; 37.2023-44.2023 ; 46.2023-49.2023 |
| Silstedt | ST | 70000 | 45.2022-50.2022 ; 02.2023-10.2023 ; 12.2023-19.2023 ; 21.2023-32.2023 ; 35.2023-43.2023 ; 44.2023-47.2023 ; 49.2023-51.2023 |
| Speyer | RP | 73215 | 40.2022-50.2022 ; 01.2023-51.2023 |
| Stadtbergen | BY | 13800 | 22.2023-22.2023 ; 24.2023-28.2023 ; 30.2023-34.2023 ; 36.2023-37.2023 ; 38.2023-52.2023 |
| Starnberg | BY | 65142 | 40.2023-52.2023 |
| Stendal | ST | 100000 | 02.2023-10.2023 ; 11.2023-19.2023 ; 20.2023-30.2023 ; 31.2023-42.2023 ; 44.2023-50.2023 ; 52.2023-52.2023 |
| Straubing | BY | 58895 | 45.2022-46.2022 ; 47.2022-50.2022 ; 52.2022-01.2023 ; 03.2023-09.2023 ; 10.2023-19.2023 ; 21.2023-51.2023 |
| Stuttgart | BW | 600000 | 45.2023-52.2023 |
| Teisendorf | BY | 8300 | 22.2022-45.2022 ; 47.2022-50.2022 ; 52.2022-52.2022 ; 02.2023-03.2023 ; 05.2023-08.2023 ; 10.2023-13.2023 ; 15.2023-32.2023 ; 33.2023-43.2023 ; 45.2023-52.2023 |
| Trier | RP | 97927 | 40.2022-51.2022 ; 01.2023-09.2023 ; 11.2023-19.2023 ; 20.2023-31.2023 ; 33.2023-35.2023 ; 38.2023-51.2023 |
| Tübingen | BW | 105000 | 46.2023-49.2023 ; 51.2023-51.2023 |
| Weiden | BY | 43754 | 40.2022-47.2022 ; 49.2022-49.2022 ; 51.2022-52.2023 |
| Weil am Rhein | BW | 103000 | 49.2023-52.2023 |
| Weißenfels | ST | 33600 | 45.2022-50.2022 ; 01.2023-02.2023 ; 03.2023-10.2023 ; 12.2023-19.2023 ; 21.2023-22.2023 ; 24.2023-51.2023 |
| Wellesweiler | SL | 44905 | 45.2023-52.2023 |
| Wiesbaden-Biebrich | HE | 130229 | 49.2023-51.2023 |
| Wiesbaden-Stadt | HE | 177681 | 49.2023-50.2023 |
| Wolfsburg | NI | 137608 | 45.2023-48.2023 ; 49.2023-52.2023 |
| Worms | RP | 78400 | 40.2022-43.2023 ; 45.2023-47.2023 ; 49.2023-52.2023 |
| Wuppertal | NW | 317483 | 22.2022-51.2022 ; 01.2023-52.2023 |
| Wustweiler | SL | 26528 | 45.2023-52.2023 |
| Zeitz | ST | 35431 | 45.2022-50.2022 ; 02.2023-10.2023 ; 12.2023-19.2023 ; 22.2023-51.2023 |
| Zusmarshausen | BY | 6375 | 51.2023-51.2023 |
| Zweibrücken | RP | 36498 | 40.2022-51.2023 |

**Table S2: Wastewater treatment plants that were part of the AMELAG study in the timeframe June 2022 to December 2023.** Location corresponds to the treatment plant location or city, federal state to the corresponding federal state (ISO 3166 coded) where the plant is located and population to the population connected to the treatment plants. The time frame column denotes the periods in which the treatment plants were sampled from (format WW.YYYY). The information where aggregated from the AMELAG dataset (https://github.com/robert-koch-institut/Abwassersurveillance_AMELAG).

| Year | Week | BB | BE | BW | BY | HB | HE | HH | MV | NI | NW | RP | SH | SL | SN | ST | TH | Plants | Population |
| --- | --- | --- | --- | --- | --- | --- | --- | --- | --- | --- | --- | --- | --- | --- | --- | --- | --- | --- | --- |
| 2022 | 22 | 0 | 1 | 1 | 9 | 1 | 0 | 2 | 0 | 0 | 12 | 1 | 0 | 0 | 1 | 0 | 1 | 29 | 11547799 |
| 2022 | 23 | 0 | 1 | 1 | 8 | 1 | 0 | 2 | 0 | 0 | 12 | 1 | 0 | 1 | 1 | 0 | 1 | 29 | 11666493 |
| 2022 | 24 | 0 | 1 | 1 | 8 | 1 | 0 | 2 | 0 | 0 | 11 | 1 | 0 | 1 | 1 | 0 | 1 | 28 | 10773788 |
| 2022 | 25 | 0 | 1 | 1 | 9 | 1 | 0 | 2 | 0 | 0 | 12 | 1 | 0 | 1 | 1 | 0 | 1 | 30 | 11671793 |
| 2022 | 26 | 0 | 1 | 1 | 8 | 1 | 0 | 2 | 0 | 0 | 12 | 1 | 0 | 1 | 1 | 0 | 1 | 29 | 11637243 |
| 2022 | 27 | 0 | 1 | 1 | 8 | 0 | 0 | 2 | 0 | 0 | 12 | 1 | 0 | 1 | 1 | 0 | 1 | 28 | 11042243 |
| 2022 | 28 | 0 | 1 | 1 | 9 | 1 | 0 | 2 | 0 | 0 | 12 | 1 | 0 | 1 | 1 | 0 | 1 | 30 | 11671793 |
| 2022 | 29 | 0 | 1 | 1 | 9 | 1 | 0 | 2 | 0 | 0 | 12 | 1 | 0 | 1 | 1 | 0 | 1 | 30 | 11671793 |
| 2022 | 30 | 0 | 1 | 1 | 9 | 1 | 0 | 2 | 0 | 0 | 12 | 1 | 0 | 1 | 1 | 0 | 1 | 30 | 11671793 |
| 2022 | 31 | 0 | 1 | 1 | 9 | 1 | 0 | 2 | 0 | 0 | 12 | 1 | 0 | 1 | 1 | 0 | 1 | 30 | 11671793 |
| 2022 | 32 | 0 | 1 | 1 | 8 | 1 | 0 | 2 | 0 | 0 | 12 | 1 | 0 | 1 | 1 | 0 | 0 | 28 | 11455607 |
| 2022 | 33 | 0 | 1 | 1 | 8 | 1 | 0 | 2 | 0 | 0 | 12 | 1 | 0 | 1 | 1 | 0 | 1 | 29 | 11571793 |
| 2022 | 34 | 0 | 1 | 1 | 8 | 1 | 0 | 2 | 0 | 0 | 12 | 1 | 0 | 1 | 1 | 1 | 1 | 30 | 11707360 |
| 2022 | 35 | 0 | 1 | 1 | 9 | 1 | 0 | 2 | 0 | 0 | 12 | 1 | 0 | 1 | 1 | 1 | 1 | 31 | 11712660 |
| 2022 | 36 | 0 | 1 | 1 | 9 | 1 | 0 | 2 | 0 | 0 | 12 | 1 | 0 | 1 | 1 | 1 | 1 | 31 | 11712660 |
| 2022 | 37 | 0 | 1 | 1 | 9 | 1 | 0 | 2 | 0 | 0 | 12 | 1 | 0 | 1 | 1 | 1 | 0 | 30 | 11596474 |
| 2022 | 38 | 0 | 1 | 1 | 9 | 1 | 0 | 2 | 0 | 0 | 12 | 1 | 0 | 1 | 1 | 1 | 1 | 31 | 11712660 |
| 2022 | 39 | 0 | 1 | 1 | 9 | 1 | 0 | 2 | 0 | 0 | 12 | 1 | 0 | 1 | 1 | 1 | 1 | 31 | 11712660 |
| 2022 | 40 | 0 | 1 | 1 | 10 | 1 | 0 | 2 | 0 | 0 | 12 | 14 | 0 | 1 | 1 | 1 | 1 | 45 | 12816648 |
| 2022 | 41 | 0 | 1 | 1 | 10 | 1 | 0 | 2 | 0 | 0 | 12 | 15 | 0 | 1 | 1 | 1 | 1 | 46 | 12844966 |
| 2022 | 42 | 0 | 1 | 1 | 11 | 1 | 0 | 2 | 0 | 0 | 12 | 16 | 0 | 1 | 1 | 1 | 1 | 48 | 13183872 |
| 2022 | 43 | 0 | 1 | 1 | 11 | 1 | 0 | 2 | 0 | 0 | 12 | 16 | 0 | 1 | 1 | 1 | 1 | 48 | 13183872 |
| 2022 | 44 | 0 | 1 | 1 | 11 | 1 | 0 | 2 | 0 | 0 | 12 | 16 | 0 | 1 | 1 | 2 | 1 | 49 | 13266872 |
| 2022 | 45 | 0 | 1 | 1 | 12 | 1 | 0 | 2 | 0 | 0 | 12 | 16 | 0 | 1 | 1 | 9 | 1 | 57 | 13855798 |
| 2022 | 46 | 0 | 1 | 1 | 12 | 1 | 0 | 2 | 0 | 0 | 12 | 16 | 0 | 1 | 1 | 9 | 1 | 57 | 13901498 |
| 2022 | 47 | 0 | 1 | 1 | 12 | 1 | 0 | 2 | 0 | 0 | 12 | 16 | 0 | 1 | 1 | 9 | 1 | 57 | 13904498 |
| 2022 | 48 | 0 | 1 | 1 | 11 | 1 | 0 | 2 | 0 | 0 | 12 | 16 | 0 | 1 | 1 | 9 | 1 | 56 | 13851144 |
| 2022 | 49 | 0 | 1 | 1 | 13 | 1 | 0 | 2 | 0 | 0 | 12 | 16 | 0 | 1 | 1 | 8 | 1 | 57 | 14038830 |
| 2022 | 50 | 0 | 1 | 1 | 13 | 1 | 0 | 2 | 0 | 0 | 12 | 16 | 0 | 1 | 1 | 8 | 1 | 57 | 13953376 |
| 2022 | 51 | 0 | 1 | 1 | 13 | 1 | 0 | 2 | 0 | 0 | 12 | 15 | 0 | 1 | 1 | 1 | 1 | 49 | 13915496 |
| 2022 | 52 | 0 | 1 | 1 | 13 | 1 | 0 | 2 | 0 | 0 | 2 | 12 | 0 | 1 | 0 | 1 | 1 | 35 | 9489450 |
| 2023 | 1 | 0 | 1 | 1 | 11 | 1 | 0 | 2 | 0 | 0 | 12 | 14 | 0 | 1 | 1 | 8 | 1 | 53 | 14403106 |
| 2023 | 2 | 0 | 1 | 1 | 14 | 1 | 0 | 2 | 0 | 0 | 12 | 15 | 0 | 1 | 1 | 12 | 1 | 61 | 15057487 |
| 2023 | 3 | 0 | 1 | 1 | 15 | 1 | 0 | 2 | 0 | 0 | 12 | 16 | 0 | 1 | 1 | 12 | 1 | 63 | 15141282 |
| 2023 | 4 | 0 | 1 | 1 | 14 | 1 | 0 | 2 | 0 | 0 | 12 | 16 | 0 | 1 | 1 | 11 | 1 | 61 | 15092982 |
| 2023 | 5 | 0 | 1 | 1 | 15 | 1 | 0 | 2 | 0 | 0 | 12 | 16 | 0 | 1 | 1 | 11 | 1 | 62 | 15101282 |
| 2023 | 6 | 0 | 1 | 1 | 15 | 1 | 0 | 2 | 0 | 0 | 12 | 16 | 0 | 1 | 1 | 10 | 1 | 61 | 14721282 |
| 2023 | 7 | 0 | 1 | 1 | 15 | 1 | 0 | 2 | 0 | 0 | 12 | 16 | 0 | 1 | 1 | 11 | 1 | 62 | 15101282 |
| 2023 | 8 | 0 | 1 | 1 | 15 | 1 | 0 | 2 | 0 | 0 | 12 | 16 | 0 | 1 | 1 | 12 | 1 | 63 | 15141282 |
| 2023 | 9 | 0 | 1 | 1 | 14 | 1 | 0 | 2 | 0 | 0 | 12 | 16 | 0 | 1 | 1 | 12 | 1 | 62 | 15132982 |
| 2023 | 10 | 0 | 1 | 1 | 15 | 1 | 0 | 2 | 0 | 0 | 12 | 15 | 0 | 1 | 1 | 10 | 1 | 60 | 14919488 |
| 2023 | 11 | 0 | 1 | 1 | 15 | 1 | 0 | 2 | 0 | 0 | 12 | 16 | 0 | 1 | 1 | 3 | 1 | 54 | 14701513 |
| 2023 | 12 | 0 | 1 | 1 | 15 | 1 | 0 | 2 | 0 | 0 | 12 | 16 | 0 | 1 | 3 | 12 | 1 | 65 | 15407648 |
| 2023 | 13 | 0 | 1 | 1 | 16 | 1 | 0 | 2 | 0 | 0 | 12 | 16 | 0 | 1 | 3 | 12 | 1 | 66 | 15461648 |
| 2023 | 14 | 0 | 1 | 1 | 14 | 1 | 0 | 2 | 0 | 0 | 12 | 16 | 0 | 1 | 3 | 12 | 0 | 63 | 15283162 |
| 2023 | 15 | 0 | 1 | 1 | 15 | 1 | 0 | 2 | 0 | 0 | 12 | 16 | 0 | 1 | 3 | 12 | 1 | 65 | 15182174 |
| 2023 | 16 | 0 | 1 | 1 | 16 | 1 | 0 | 2 | 0 | 0 | 12 | 16 | 0 | 1 | 3 | 12 | 1 | 66 | 15461648 |
| 2023 | 17 | 0 | 1 | 1 | 16 | 1 | 0 | 2 | 0 | 0 | 12 | 15 | 0 | 1 | 3 | 12 | 0 | 64 | 15205462 |
| 2023 | 18 | 0 | 1 | 1 | 16 | 1 | 0 | 2 | 0 | 0 | 12 | 16 | 0 | 1 | 3 | 12 | 1 | 66 | 15461648 |
| 2023 | 19 | 0 | 1 | 1 | 15 | 1 | 0 | 2 | 1 | 0 | 12 | 16 | 0 | 1 | 3 | 9 | 1 | 63 | 14950068 |
| 2023 | 20 | 0 | 1 | 1 | 14 | 0 | 0 | 2 | 1 | 0 | 11 | 16 | 0 | 1 | 3 | 4 | 1 | 55 | 13506342 |
| 2023 | 21 | 0 | 1 | 1 | 16 | 1 | 0 | 2 | 1 | 0 | 12 | 16 | 0 | 1 | 3 | 11 | 1 | 66 | 15666637 |
| 2023 | 22 | 1 | 1 | 1 | 18 | 1 | 0 | 2 | 1 | 0 | 12 | 16 | 0 | 1 | 3 | 12 | 1 | 70 | 15821968 |
| 2023 | 23 | 1 | 1 | 1 | 17 | 1 | 0 | 2 | 1 | 0 | 12 | 14 | 0 | 1 | 3 | 11 | 1 | 66 | 15699268 |
| 2023 | 24 | 1 | 1 | 1 | 18 | 1 | 0 | 2 | 1 | 0 | 12 | 16 | 0 | 1 | 3 | 12 | 1 | 70 | 15821968 |
| 2023 | 25 | 1 | 1 | 1 | 18 | 1 | 0 | 2 | 1 | 0 | 11 | 16 | 0 | 1 | 3 | 11 | 1 | 68 | 14621513 |
| 2023 | 26 | 1 | 1 | 1 | 17 | 1 | 0 | 2 | 1 | 0 | 12 | 16 | 0 | 1 | 3 | 12 | 1 | 69 | 15542494 |
| 2023 | 27 | 1 | 1 | 2 | 18 | 1 | 0 | 2 | 1 | 0 | 12 | 16 | 0 | 1 | 3 | 12 | 1 | 71 | 15849207 |
| 2023 | 28 | 1 | 1 | 3 | 17 | 1 | 0 | 2 | 1 | 0 | 12 | 15 | 0 | 1 | 3 | 12 | 0 | 69 | 15325332 |
| 2023 | 29 | 1 | 1 | 3 | 17 | 1 | 0 | 2 | 1 | 0 | 12 | 15 | 0 | 1 | 3 | 12 | 1 | 70 | 15807718 |
| 2023 | 30 | 1 | 1 | 3 | 18 | 1 | 0 | 2 | 1 | 0 | 12 | 16 | 0 | 1 | 3 | 11 | 1 | 71 | 15585518 |
| 2023 | 31 | 1 | 1 | 3 | 18 | 1 | 0 | 2 | 1 | 0 | 12 | 16 | 0 | 1 | 3 | 13 | 1 | 73 | 15946518 |
| 2023 | 32 | 1 | 1 | 3 | 19 | 1 | 0 | 2 | 1 | 0 | 12 | 15 | 0 | 1 | 3 | 12 | 1 | 72 | 15996591 |
| 2023 | 33 | 1 | 1 | 3 | 19 | 1 | 0 | 2 | 1 | 0 | 12 | 16 | 0 | 1 | 3 | 12 | 0 | 72 | 15989762 |
| 2023 | 34 | 1 | 1 | 3 | 19 | 1 | 0 | 2 | 1 | 0 | 12 | 16 | 0 | 1 | 3 | 12 | 1 | 73 | 16096518 |
| 2023 | 35 | 1 | 1 | 3 | 19 | 1 | 0 | 2 | 1 | 0 | 12 | 16 | 0 | 1 | 3 | 13 | 1 | 74 | 16171218 |
| 2023 | 36 | 1 | 1 | 3 | 20 | 1 | 0 | 2 | 1 | 0 | 12 | 15 | 0 | 1 | 3 | 13 | 1 | 74 | 16087091 |
| 2023 | 37 | 1 | 1 | 3 | 20 | 1 | 0 | 2 | 1 | 0 | 12 | 15 | 0 | 1 | 3 | 13 | 1 | 74 | 16087091 |
| 2023 | 38 | 1 | 1 | 3 | 20 | 1 | 0 | 2 | 1 | 0 | 12 | 16 | 0 | 1 | 3 | 13 | 1 | 75 | 16185018 |
| 2023 | 39 | 1 | 1 | 3 | 22 | 1 | 0 | 2 | 1 | 0 | 12 | 16 | 0 | 1 | 3 | 13 | 1 | 77 | 16380609 |
| 2023 | 40 | 1 | 1 | 3 | 23 | 1 | 0 | 2 | 1 | 0 | 12 | 16 | 0 | 1 | 3 | 13 | 1 | 78 | 16445751 |
| 2023 | 41 | 1 | 1 | 3 | 25 | 1 | 0 | 2 | 1 | 0 | 12 | 16 | 0 | 1 | 4 | 13 | 1 | 81 | 16700174 |
| 2023 | 42 | 1 | 1 | 1 | 25 | 1 | 0 | 2 | 1 | 0 | 12 | 16 | 0 | 1 | 4 | 13 | 1 | 79 | 16647624 |
| 2023 | 43 | 1 | 1 | 1 | 25 | 1 | 0 | 2 | 1 | 0 | 12 | 16 | 0 | 0 | 4 | 12 | 1 | 77 | 16423630 |
| 2023 | 44 | 1 | 1 | 1 | 15 | 1 | 0 | 2 | 1 | 0 | 14 | 15 | 0 | 0 | 4 | 13 | 1 | 69 | 16138023 |
| 2023 | 45 | 4 | 1 | 9 | 28 | 1 | 5 | 2 | 3 | 7 | 14 | 15 | 6 | 4 | 4 | 12 | 1 | 116 | 22668818 |
| 2023 | 46 | 4 | 1 | 10 | 24 | 1 | 5 | 2 | 4 | 7 | 14 | 16 | 6 | 4 | 4 | 13 | 1 | 116 | 22558666 |
| 2023 | 47 | 4 | 1 | 10 | 27 | 1 | 6 | 2 | 4 | 9 | 14 | 16 | 7 | 4 | 4 | 13 | 1 | 123 | 23619914 |
| 2023 | 48 | 4 | 1 | 15 | 28 | 1 | 5 | 2 | 4 | 9 | 13 | 15 | 7 | 4 | 4 | 12 | 1 | 125 | 23250865 |
| 2023 | 49 | 4 | 1 | 16 | 27 | 1 | 9 | 2 | 4 | 9 | 14 | 16 | 8 | 4 | 4 | 13 | 1 | 133 | 25061603 |
| 2023 | 50 | 4 | 1 | 15 | 28 | 1 | 7 | 2 | 4 | 9 | 14 | 16 | 8 | 4 | 4 | 12 | 0 | 129 | 23647654 |
| 2023 | 51 | 4 | 1 | 16 | 29 | 1 | 5 | 2 | 4 | 9 | 11 | 16 | 8 | 4 | 3 | 9 | 0 | 122 | 20868732 |
| 2023 | 52 | 0 | 1 | 15 | 24 | 1 | 0 | 2 | 0 | 9 | 4 | 11 | 8 | 4 | 3 | 7 | 0 | 89 | 14463775 |

**Table S3: Wastewater treatment plants counts per week and federal state that were part of the AMELAG study in the timeframe June 2022 to December 2023.** Plants corresponds to the sum over all federal state plants (country level) and population to the total population connected to these plants. The information where aggregated from the AMELAG dataset (https://github.com/robert-koch-institut/Abwassersurveillance_AMELAG).

| Model | Hyperparameter | Search Space | Sampler |
| --- | --- | --- | --- |
| Random Forest | Min_samples_split Min_samples_leaf | [2, 4, 8, 16] [1, 2, 4] | Grid-Search |
| XGBoost | Learning rate Max_depth  Reg_lambda Reg_alpha | (0.01, 0.5) (2, 12) (0, 10) (0, 10) | TPE |

**Table S4: Hyperparameters for Random Forest and XGBoost models.** The brackets correspond to discrete values, while the parentheses correspond to continuous parameter ranges. Hyperparameters were either optimized by using a grid-search or with a tree-structured Parzen estimator from optuna. As the model’s hyperparameters were tuned for each context window individually we did not provide the final choices.
